# Soluble urokinase plasminogen activator receptor (suPAR) as a prognostic biomarker in acutely admitted patients with atrial fibrillation

**DOI:** 10.1002/joa3.70077

**Published:** 2025-04-23

**Authors:** Frederik Dencker Wisborg, Nora Olsen El Caidi, Ida Arentz Taraldsen, Sandra Tonning, Aginsha Kandiah, Mohammed El‐Sheikh, Hashmat S. Z. Bahrami, Ove Andersen, Line Jee Hartmann Rasmussen, Jens Hove, Ulrik Dixen, Johannes Grand

**Affiliations:** ^1^ Department of Cardiology Copenhagen University Hospital, Amager and Hvidovre Hvidovre Denmark; ^2^ Department of Clinical and Translational Research Steno Diabetes Center Copenhagen Herlev Denmark; ^3^ Department of Clinical Research Copenhagen University Hospital, Amager and Hvidovre Hvidovre Denmark; ^4^ Department of Emergency Medicine Copenhagen University Hospital, Amager and Hvidovre Hvidovre Denmark; ^5^ Faculty of Health and Medical Sciences University of Copenhagen Copenhagen Denmark

**Keywords:** acute cardiovascular care, arrrhythmia, atrial fibrillation, mortality, suPAR

## Abstract

**Background:**

Atrial fibrillation (AF) is associated with a higher incidence of stroke, heart failure, and mortality. Risk assessment of clinical outcomes in patients hospitalized acutely with AF remains a challenge.

**Purpose:**

To investigate if soluble urokinase plasminogen activator receptor (suPAR) levels at admission to the Emergency Department (ED) are associated with 1‐year all‐cause mortality in patients admitted with AF.

**Methods:**

A prospective cohort study of patients consecutively admitted to the medical ED of a university hospital in Copenhagen, Denmark, between 2020 and 2022 with symptoms of COVID‐19. Patients were included if they were admitted with AF as the primary or secondary diagnosis. All patients had suPAR measured at the index admission, and follow‐up was up to 1 year. The association between suPAR and 1‐year mortality was investigated with multivariate Cox regression. We adjusted for age, sex, smoking, C‐reactive protein, creatinine, hemoglobin, albumin, and comorbidities.

**Results:**

Of the 7,258 patients included during the period, 362 (5.0%) patients were admitted with AF as the primary or secondary diagnosis. Due to missing data, 23 (6.4%) patients were excluded. Among the remaining 339 patients, 68 (20.1%) patients were dead at follow‐up. The multivariate Cox regression showed that elevated suPAR was independently associated with an increased risk of 1‐year mortality, with a hazard ratio of 1.12 (95% confidence interval: 1.05–1.20, *p* < 0.001).

**Conclusion:**

Elevated suPAR levels were significantly associated with 1‐year all‐cause mortality in patients acutely admitted with AF to the ED.

## Graphical abstract

In an observational study with 339 patients admitted acutely to an Emergency Department with atrial fibrillation, elevated levels of the inflammatory biomarker, soluble urokinase plasminogen activator receptor (suPAR), measured upon admission, were significantly associated with 1‐year all‐cause mortality. Kaplan–Meier plot showing the association between suPAR, stratified according to intervals (≤4ng/mL, 4–6ng/mL, ≥6 ng/mL, and survival probability). suPAR, soluble urokinase plasminogen activator receptor.

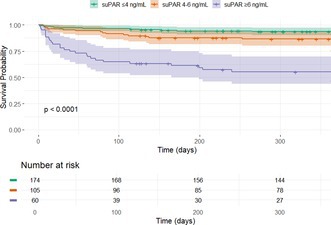

## 1 INTRODUCTION

Atrial fibrillation (AF) is the most prevalent, sustained arrhythmia in the world,^1^ with a prevalence of about 2%,^2^ causing a substantial economic and social burden.^3^, ^4^ The clinical manifestations of AF demonstrate vast heterogeneity, ranging from an asymptomatic state to severe complications such as ischemic stroke, congestive heart failure, and death.^5^, ^6^, ^7^ Despite advances in rhythm and rate control treatments, one‐third of patients with AF ultimately die due to heart failure.^8^

Patients with AF are frequently hospitalized in the Emergency Department (ED),^9^ where treatment options include direct current (DC) cardioversion, antiarrhythmic drug therapy, and management of derived conditions, such as heart failure or stroke. Prognostic assessment in AF patients often requires a multifactorial approach, and biomarkers may aid in risk stratification and treatment planning.

While the exact triggers of a paroxysm of AF are not fully understood, several risk factors have been identified, including advanced age, cardiopulmonary disease, and lifestyle factors.^10^, ^11^ Chronic inflammation may play a role in the pathogenesis of AF.^12^

Soluble urokinase plasminogen activator receptor (suPAR) is an inflammatory marker that can be measured in blood,^13^ urine,^13^ saliva,^14^ and cerebrospinal fluid.^15^ suPAR is the soluble form of the membrane‐bound urokinase plasminogen activator receptor (uPAR) primarily expressed on the surface of immune cells, endothelial cells, and smooth muscle cells.^16^ As a biomarker of low‐grade chronic systemic inflammation,^16^ suPAR is associated with a wide range of systemic disorders and diseases^17^, ^18^, ^19^, ^20^, ^21^ and predicts disease severity and mortality.^22^, ^23^ Furthermore, suPAR has been shown to outperform C‐reactive protein (CRP) in predicting various cardiovascular events.^17^, ^24^

This study aimed to investigate whether suPAR was associated with clinical outcomes in patients acutely admitted with AF to the ED.

## 2 METHODS

### 2.1 Study design and population

This study is based on a prospective cohort of adult (≥18 years old) patients with symptoms of COVID‐19 consecutively admitted to the ED of Copenhagen University Hospital, Amager and Hvidovre (AHH), Hvidovre, between March 10, 2020, and March 31, 2022. AHH serves 570,000 inhabitants. Adult patients from all internal medicine specialties are treated in this ED, excluding those assumed to have gastroenterological, gynecological, or obstetrical conditions.

Research staff collected blood samples (within the first 2 hours after admission) in the ED during the inclusion period. These included suPAR and a standard panel of admission blood tests: leucocytes, neutrophilocytes, lymphocytes, hemoglobin, thrombocytes, creatinine, sodium, potassium, urea, albumin, alanine transaminase, lactate dehydrogenase, bilirubin, and CRP. All patients had vital signs measured upon admission, including pulse, systolic and diastolic blood pressure, respiratory frequency, peripheral oxygen saturation (pulse oximetry), oxygen supplementation, body temperature, and level of consciousness.

Patients were included if they were admitted with AF as the primary or secondary diagnosis during the index admission. Follow‐up was up to 1 year, with a minimum duration of 90 days.

Index admission was defined as the first admission in which suPAR was measured. Each patient's unique civil registration number was used to extract data on biochemistry via the electronic hospital database, diagnoses and dates of hospital admission and discharge from the Danish National Patient Registry, and information on sex, birth date, and vital status at the end of follow‐up from the Danish Civil Registration System.

Study data were managed using the Research Electronic Data Capture (REDCap) tool hosted by the Capital Region of Denmark.

### 2.2 suPAR analysis

Plasma suPAR levels were measured in singlets using the suPARnostic AUTO Flex ELISA (Virogates A/S, Birkerød, Denmark) following the manufacturer's instructions at the research laboratories at the Department of Clinical Research, AHH.

### 2.3 Outcomes

The primary outcome was 1‐year all‐cause mortality. Secondary outcomes were length of in‐hospital stay (index admission), readmission count (unplanned, acute readmissions) within 1 year from index admission, and admission to the intensive care unit (ICU) within 1 year from the index admission.

### 2.4 Statistics

Continuous and categorical variables are presented as median (interquartile range [IQR]) and *n* (%), respectively. Continuous variables were tested for normality with the Andersson–Darling test. Differences between groups were tested with the analysis of variance or the Kruskal–Wallis test for parametric and nonparametric variables, respectively. Differences between categorical variables were tested with the chi‐square test.

A Kaplan–Meier plot was produced to illustrate survival probability based on suPAR levels, with patients stratified into the following groups: ≤4, 4–6, and ≥6 ng/mL.^25^, ^26^ The group with suPAR levels ≤4 ng/mL was defined as “low suPAR,” the group with suPAR levels 4–6 ng/mL was defined as “intermediate suPAR,” and the group with suPAR levels ≥6 ng/mL was defined as “high suPAR.” A spline plot was produced to demonstrate the probability of 1‐year mortality with suPAR displayed as a continuous variable.

Multivariate Cox regression analyses were performed to estimate the association between suPAR and 1‐year mortality. CRP and creatinine were logarithm‐transformed (natural logarithm) due to nonnormal distribution. In model 1, we adjusted for age, sex, smoking, ln(CRP), and ln(creatinine). In model 2, we adjusted for the same covariates as in model 1 and included additional adjustments for comorbidities (type 2 diabetes mellitus, hypertension, congestive heart failure, stroke, chronic obstructive pulmonary disease (COPD), and ischemic heart disease), hemoglobin, and albumin. In model 3, suPAR was stratified by the defined intervals (low suPAR, intermediate suPAR, and high suPAR), and we adjusted for the same covariates as in model 1. Results are presented as hazard ratios (HRs) with 95% confidence intervals (CIs).

Additionally, to assess the predictive performance of various variables for 1‐year mortality, we performed a receiver‐operating characteristic (ROC) curve analysis and calculated the corresponding area under the curve (AUC). Thus, AUCs were calculated for the combination of age and CRP, as these covariates were identified as independent risk factors for 1‐year mortality in the Cox regression analysis. Subsequently, we added suPAR to test for the superiority of this combination compared with the former.

For the secondary outcomes, we used logistic regression to assess the binary outcome (readmission to the ICU) and Poisson regression to assess numerical outcomes (length of in‐hospital stay and readmission count). The models were adjusted for sex, age, smoking, CRP, and creatinine.

For all analyses, a two‐sided *p* < 0.05 was considered to be statistically significant. The program R 4.4.1 (R Foundation for Statistical Computing, Vienna, Austria) was used for analyses and figures.

### 2.5 Ethics

The study was approved by the Danish Data Protection Agency (record number P‐2020‐513) and by the Patient Safety Authorities (record number 31–1521‐319) allowing the use of anonymized registry‐based data for research purposes without informed consent. During admission, patients received treatment as usual, and this observational study did not cause any delay or intervention in their course of admission.

## 3 RESULTS

### 3.1 Baseline characteristics

**FIGURE 1:**
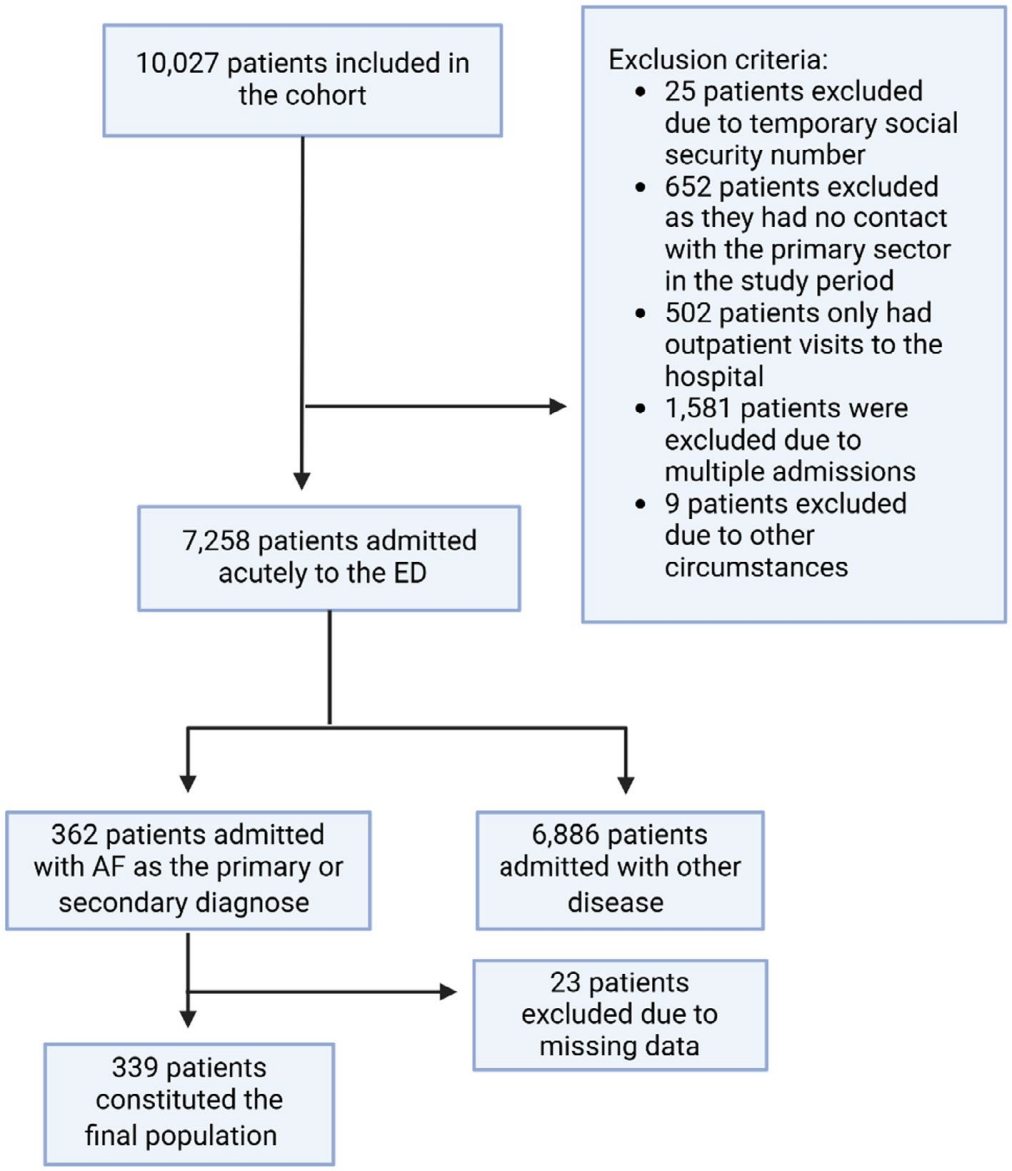
Flow chart of patient inclusion and exclusion.

A total of 10,027 patients were included in the cohort between March 10, 2020, and March 31, 2022, of which 2,769 patients were excluded (Figure 1). Out of the remaining 7,258 patients, 362 (5,0%) were admitted with AF as the primary or secondary diagnosis. Of these, 23 (6.4%) patients were excluded due to missing data, leaving 339 patients for the final population. The median age was 73 years (IQR 63–82), and 166 (49.0%) patients were women. suPAR levels were roughly normally distributed with a median of 4.00 ng/mL (IQR: 3.00–5.25 ng/mL) and a mean of 4.61 ng/mL (Figures S1 and S2).

Baseline data, stratified according to suPAR intervals (≤4, 4–6 and ≥6 ng/mL), are shown in Table 1. The groups differed from each other on several baseline parameters, including age, smoking, hemoglobin, albumin, CRP, creatinine, leukocytes, oxygen supply, peripheral oxygen saturation (pulse oximetry), body temperature, respiratory frequency, and prevalence of heart failure and COPD.

**TABLE 1:** Baseline characteristics.

	suPAR ≤4 ng/mL (*N* = 174)	suPAR 4–6 ng/mL (*N* = 105)	suPAR ≥6 ng/mL (*N* = 60)	All patients (*N* = 339)	*p*‐value
Age (years)	67.0 (58.0–77.0)	76.0 (68.0–84.0)	79.0 (70.5–87.0)	73.0 (63.0–82.0)	<0.001
Sex (women)	82 (47.1%)	56 (53.3%)	28 (46.7%)	166 (49.0%)	0.56
Smoking					
Previous	74 (42.5%)	46 (43.8%)	39 (65.0%)	159 (46.9%)	0.015
Current	25 (14.4%)	21 (20.0%)	8 (13.3%)	54 (15.9%)	
Excessive alcohol consumption^a^					
Quit	19 (10.9%)	14 (13.3%)	13 (21.7%)	46 (13.6%)	0.29
Yes	89 (51.1%)	51 (48.6%)	24 (40.0%)	164 (48.4%)	
IHD (yes)	34 (19.5%)	17 (16.2%)	17 (28.3%)	68 (20.1%)	0.17
HF (yes)	23 (13.2%)	29 (27.6%)	23 (38.3%)	75 (22.1%)	<0.001
DM2 (yes)	16 (9.2%)	5 (4.8%)	8 (13.3%)	29 (8.6%)	0.15
HT (yes)	57 (32.8%)	44 (41.9%)	24 (40.0%)	125 (36.9%)	0.27
COPD (yes)	10 (5.7%)	16 (15.2%)	11 (18.3%)	37 (10.9%)	0.006
Stroke (yes)	14 (8.0%)	12 (11.4%)	8 (13.3%)	34 (10.0%)	0.43
suPAR (ng/mL)	3.0 (2.4–3.5)	4.7 (4.4–5.2)	7.8 (6.5–9.6)	4.0 (3.0–5.2)	<0.001
CRP (mg/L)	2.4 (0.9–7.1)	7.7 (2.5–21.0)	28.5 (7.6–73.5)	5.3 (1.4–19.0)	<0.001
Hemoglobin (mmol/L)	8.8 (8.2–9.4)	8.3 (7.3–8.9)	7.6 (6.8–8.0)	8.5 (7.6–9.1)	<0.001
Leukocytes (10^9^/L)	7.4 (6.3–9.3)	8.7 (6.9–10.2)	10.0 (7.2–13.0)	8.1 (6.5–10.2)	<0.001
Creatinine (μmol/L)	77.0 (66.2–92.8)	89.0 (69.0–105.0)	110.5 (84.8–163.5)	83.0 (68.0–104.5)	<0.001
ALAT (U/L)	25.5 (19.0–37.8)	21.0 (15.0–31.0)	22.5 (16.0–31.5)	24.0 (17.0–34.0)	0.051
Albumin (g/L)	36.0 (33.0–37.0)	34.0 (30.0–36.0)	30.0 (27.0–34.0)	34.0 (31.0–37.0)	<0.001
Systolic BP (mm Hg)	139.5 (125.0–154.0)	141.0 (128.0–155.0)	136.0 (116.5–168.0)	139.0 (124.0–156.0)	0.55
Diastolic BP (mm Hg)	87.0 (74.2–100.8)	86.0 (75.0–100.0)	77.0 (66.8–98.5)	86.0 (73.0–100.0)	0.18
Oxygen supply (L/min) (mean)	0.32 (1.32)	1.09 (3.61)	2.15 (3.90)	0.90 (2.82)	<0.001
Ventricular rate	99 (78–140)	95 (80–128)	105 (87–140)	100 (79–134)	0.47
RF	18.0 (16.0–19.0)	19.0 (17.0–22.0)	20.0 (18.0–24.0)	18.0 (16.0–20.0)	<0.005
O_2_ saturation (%)	98.0 (96.0–99.0)	97.0 (96.0–99.0)	96.5 (95.0–98.0)	97 (96.0–99.0)	<0.001
Temperature (°C)	36.5 (36.3–36.8)	36.6 (36.4–37.0)	36.8 (36.5–37.0)	36.6 (36.4–36.9)	0.002
Length of in‐hospital stay (days)	1.0 (0.0–3.0)	4.0 (1.0–7.0)	7.0 (3.0–13.8)	2.0 (1.0–6.0)	<0.001
Readmission count	1.0 (0.0–2.0)	1.0 (1.0–3.0)	1.0 (0.0–2.0)	1.0 (0.0–3.0)	0.043
Admission to the ICU (yes)	5 (2.9%)	5 (4.8%)	7 (11.7%)	17 (5.0%)	0.026

*Note*: Data are presented as median (interquartile range), number (%), or mean (standard deviation).

Abbreviations: ALAT, alanine aminotransferase; BP, blood pressure; COPD, chronic obstructive lung disease; CRP, C‐reactive protein; DM2, diabetes mellitus type 2; HF, heart failure; HT, hypertension; ICU, intensive care unit (admission within a year from index admission).; IHD, ischemic heart disease; O_2_ saturation, peripheral oxygen saturation (pulse oximetry); RF, respiratory frequency; suPAR, soluble urokinase plasminogen activator receptor.

^a^ Excessive alcohol consumption is defined as ≥11 units/week.

### 3.2 Mortality

**FIGURE 2:**
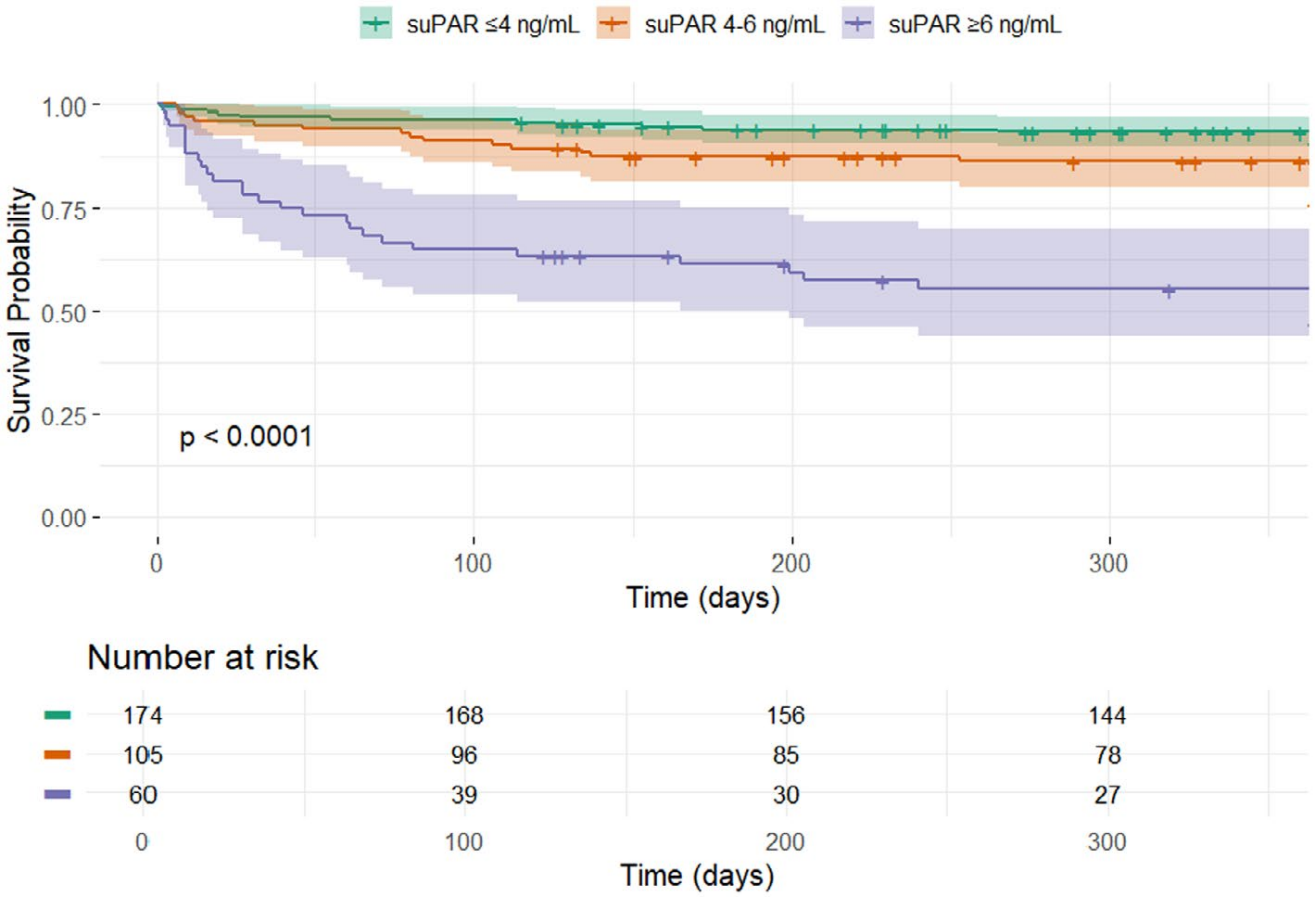
Kaplan–Meier plot showing the association between suPAR, stratified according to the defined intervals, and survival probability. suPAR, soluble urokinase plasminogen activator receptor.

**FIGURE 3:**
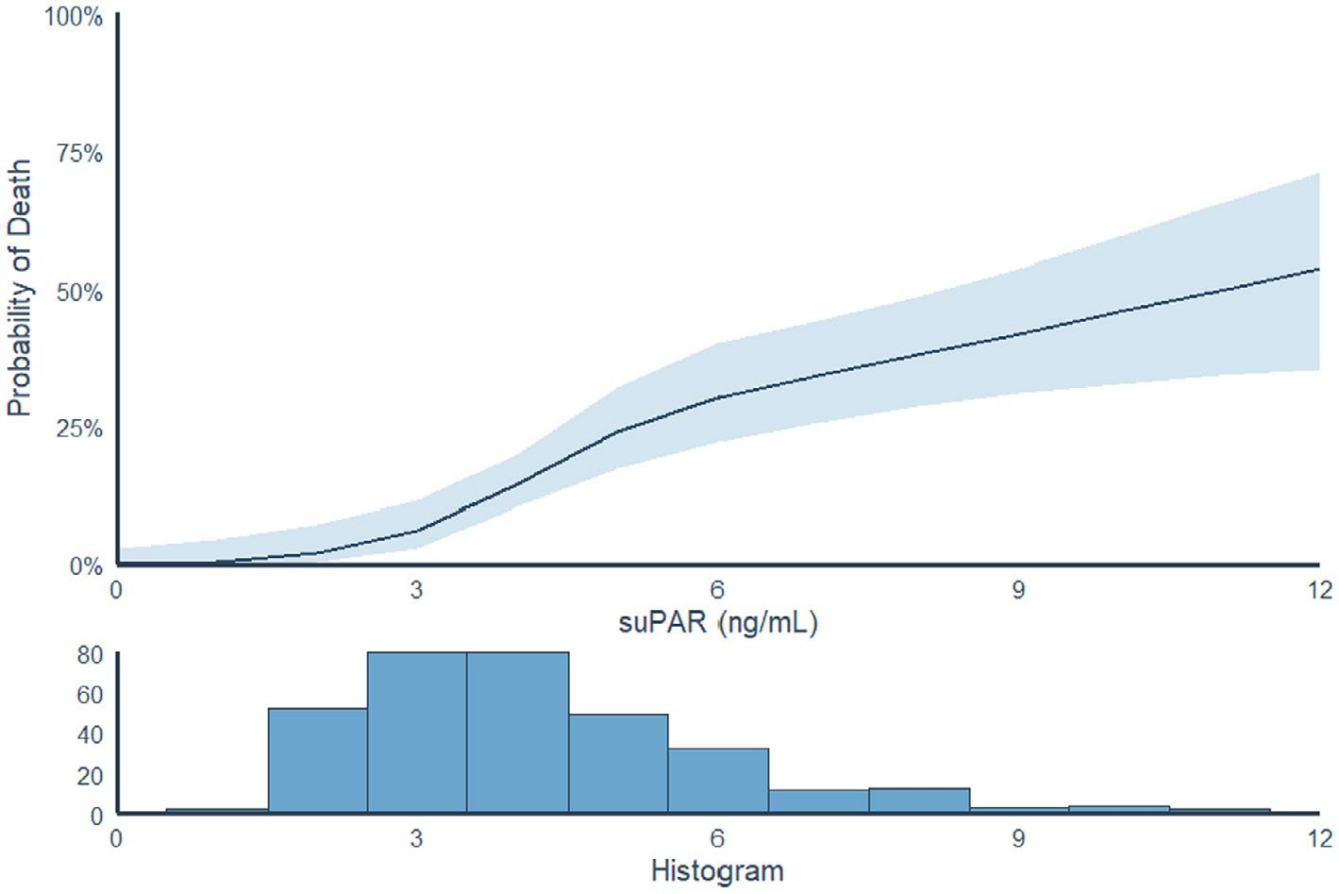
Combined spline plot and histogram showing the association between suPAR levels and the probability of mortality at follow‐up. suPAR, soluble urokinase plasminogen activator receptor.

Among the 339 patients admitted with AF, a total of 68 (20.1%) patients were dead at follow‐up; 15 out of 174 (8.6%) patients with low baseline suPAR (≤4 ng/mL), 23 out of 105 (21.9%) patients with intermediate baseline suPAR (4–6 ng/mL), and 30 out of 60 (50%) patients with high baseline suPAR (≥6 ng/mL) were dead at follow‐up. In a time‐to‐event analysis, survival probability was highest in patients with suPAR <4 ng/mL and lowest in those patients with suPAR ≥6 ng/mL (Figure 2). Using patients with low baseline suPAR as the reference group, the unadjusted HR for all‐cause mortality was 2.70 (95% CI: 1.40–5.20, *p* = 0.002) in patients with intermediate suPAR and 8.40 (95% CI: 4.50–15.60, *p* < 0.001) in those with high suPAR (Figure S3). There was a trend of increasing mortality across the full spectrum of elevated suPAR levels (Figure 3).

In the multivariate Cox regression analysis, suPAR was independently associated with 1‐year mortality in patients admitted with AF. Thus, when adjusted for age, sex, smoking, ln(CRP), and ln(creatinine), the HR for suPAR for 1‐year mortality was 1.12 (95% CI: 1.05–1.20, *p* < 0.001) (Figure S4), meaning for every unit (ng/mL) increase in suPAR, the risk of death in 1 year increased by 12%. This result remained essentially unaltered after further adjustment for comorbidities, hemoglobin, and albumin (HR 1.10, 95% CI: 1.01–1.20, *p* = 0.024) (Figure S5). Other significant risk factors included age (HR: 1.06 95% CI: 1.04–1.10, *p* < 0.001) and ln(CRP) (HR: 1.55, 95% CI: 1.29–1.90, *p* < 0.001) (Figure S4). When comparing patients with high suPAR (≥6 ng/mL) to patients with low suPAR (≤4 ng/mL), the HR for 1‐year mortality was 3.57 (95% CI: 1.70–7.50, *p* < 0.001) after adjusting for age, sex, smoking, ln(CRP), and ln(creatinine) (Figure S6).

**FIGURE 4:**
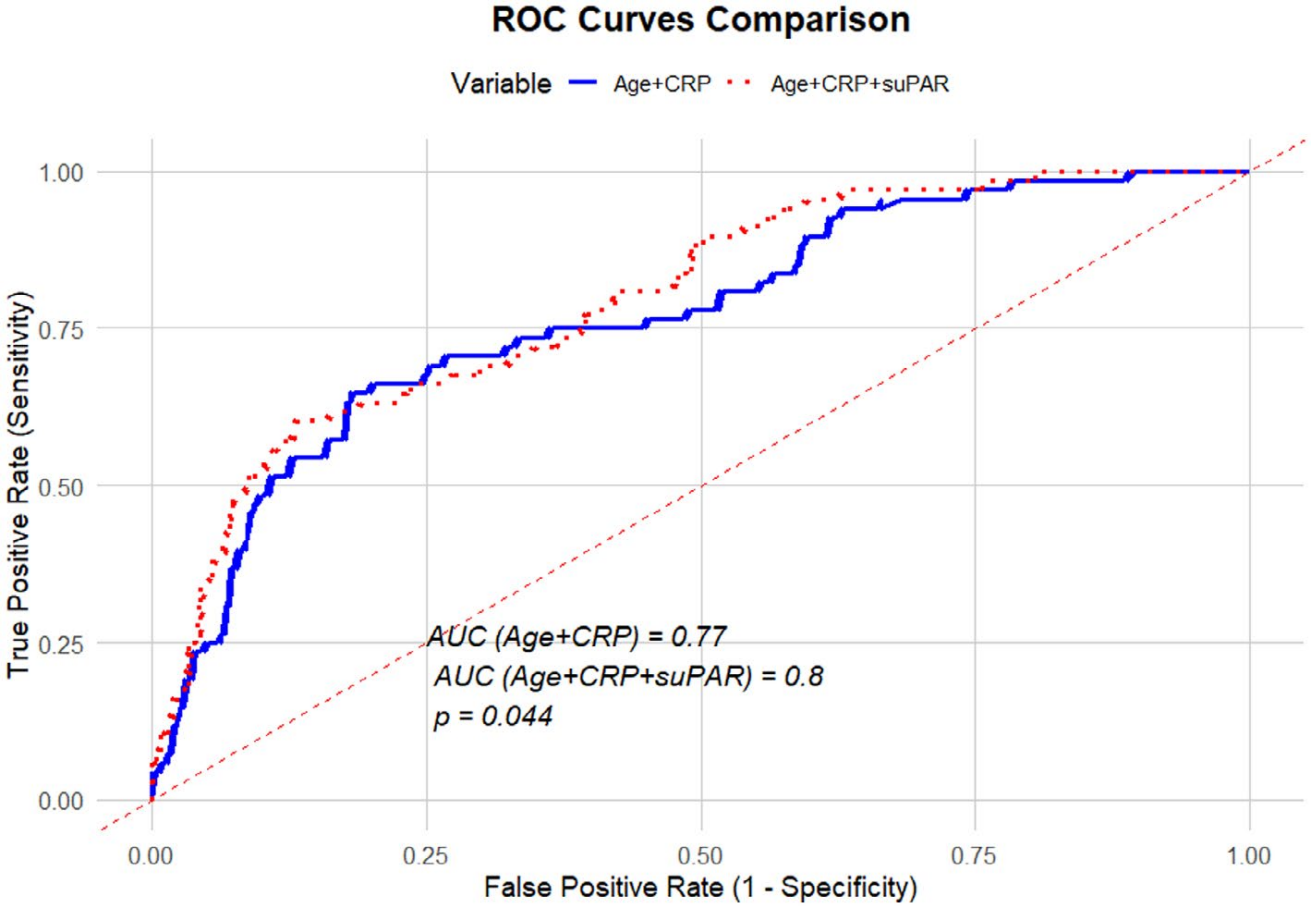
Receiver‐operating characteristics (ROC) curves showing the performance of the combination of age + CRP and age + CRP + suPAR, respectively, in predicting 1‐year mortality at follow‐up. CRP, C‐reactive protein; suPAR, soluble urokinase plasminogen activator receptor.

In the ROC analysis to predict 1‐year mortality, the AUC for suPAR was 0.77, which was slightly better than CRP (0.73); however, the difference was nonsignificant (*p* = 0.344) (Figure S7). In the multivariate analysis, the combination of age and CRP resulted in an AUC of 0.77. The further addition of suPAR to the model significantly increased the performance of the model (AUC: 0.80 vs. 0.77, *p* = 0.039) (Figure 4).

### 3.3 Secondary outcomes

Higher admissions suPAR levels were associated with increased length of in‐hospital stay. The median length of stay was 1 day (IQR: 0–3) for patients with low suPAR, 4 days (IQR: 1–7) for patients with intermediate suPAR, and 7 days (IQR: 1–14) for patients with high suPAR (*p* < 0.001). A Poisson regression model adjusted for age, sex, CRP, and creatinine showed a 7.9% increase in length of in‐hospital stay per unit increase in suPAR (ng/mL), *p* < 0.001. However, the covariates age, sex, smoking, CRP, and creatinine were also associated with length of in‐hospital stay (Figure S8).

240 out of 339 patients (70.8%) experienced at least one readmission within 1 year after discharge from the index admission. The median number of readmissions was 1.0 in all three suPAR groups (Table 1), and a Poisson regression model, adjusted for age, sex, CRP, and creatinine, showed no statistically significant association between suPAR and readmission (*p* = 0.791) (Figure S9).

The percentage of patients admitted to the ICU within 1 year from index admission was significantly associated with increasing suPAR; 5 patients (2.9%) with low suPAR, 5 patients (4.8%) with intermediate suPAR, and 7 patients (11.7%) with high suPAR admitted to the ICU (*p* = 0.026). However, a binary logistic regression model, adjusted for age, sex, CRP, and creatinine, showed no statistically significant association between suPAR and the risk of admission to the ICU (*p* = 0.42) (Figure S10). Admission to the ICU during the index hospitalization was not considered a readmission.

## 4 DISCUSSION

In a cohort of patients acutely admitted to the ED with AF, we showed that suPAR measured upon hospital arrival was independently associated with 1‐year mortality. After adjustment for other factors, an increase in suPAR of 1 ng/mL was associated with a 12% increase in the risk of 1‐year all‐cause mortality. Furthermore, individuals with high suPAR levels (≥6 ng/mL) at admission had an adjusted 3.5 times higher risk of death at follow‐up compared to individuals with low suPAR levels (≤4 ng/mL). The AUC for the combination of CRP and age in predicting 1‐year mortality was 0.77. The addition of suPAR resulted in a modest but significant improvement in the prediction of 1‐year mortality, increasing the AUC from 0.77 to 0.80, *p* = 0.039. Additionally, elevated suPAR levels were associated with prolonged hospitalization and admission to the ICU. However, the association between elevated suPAR levels and an increased rate of ICU admissions was not statistically significant after adjustment for relevant covariates. Moreover, no association was observed between suPAR levels and the risk of admission within 1 year from the index admission.

To our knowledge, this study is the first to report on the association between suPAR and mortality in patients acutely hospitalized with AF. The association between suPAR and mortality in outpatients with AF has been investigated in one previous study with similar results as ours; Pol et al.^24^ examined the association between 268 different biomarkers and cardiovascular (CV) death based on the large ARISTOLE and RE‐LY cohorts of patients with AF (around 18,000 outpatients in each studies). They identified ten biomarkers significantly associated with cardiovascular death, one of them being suPAR with a HR of 1.62 (95% CI: 1.30–2.03, *p* < 0.001), even after adjustment for additional covariates (including Cystatin C, high sensitivity Troponin T (hs‐TnT), and N‐terminal pro b‐type natriuretic peptide (NT‐proBNP)). These findings support our results despite the differences in the study designs and populations.

Despite medical advances in the treatment, AF remains a significant global cause of morbidity and mortality. In the prognostic evaluation of stroke and bleeding, the CHA2DS2‐VA (congestive heart failure, hypertension, age ≥75 years, diabetes mellitus, previous stroke or transient ischemic attack, vascular disease, age 65–74 years) and HAS‐BLED (hypertension, abnormal liver/renal function, stroke, bleeding, labile international normalized ratio's, elderly [≥65 years], drugs) scores are used, respectively.^27^ However, no similar tool exists for predicting no‐stroke complications. The Charlson Comorbidity Index (CCI) from 1987 and other later reweighted indices are measures used to estimate mortality risk. However, they only take ICD‐10 coded comorbidities into account^28^ and require exhaustive information on the patient's disease burden, which is not always available in an acute hospital setting. By contrast, inflammatory biomarkers, accessible to the clinicians during acute hospitalizations, may help predict adverse events and non‐stroke‐related mortality, thus enabling more personalized treatment strategies.

In recent years, biomarkers such as hs‐TnT,^29^ NT‐proBNP,^30^ and growth/differentiation factor 15 (GDF‐15)^31^ have been associated with adverse outcomes in patients with AF. suPAR differs from those biomarkers in several ways. hs‐TnT, NT‐proBNP, and to a lesser extent GDF‐15—which also is associated with aging,^32^ malnutrition,^33^ and inflammation^34^—are primarily correlated with cardiovascular pathology. suPAR is nonspecifically associated with a wide range of diseases.^17^, ^18^, ^19^, ^20^, ^21^ For the same reason, suPAR offers limited diagnostic utility. Additionally, suPAR's half‐life, estimated at 7–10 days,^35^, ^36^ is significantly longer than the half‐lives of cytokines, acute‐phase reactants, hs‐TnT, NT‐proBNP, and GDF‐15, which span from minutes to hours. This prolonged stability makes suPAR less susceptible to short‐term influences during acute events.^37^

suPAR's stability might limit its utility in acutely ill patients and the monitoring of the immediate clinical response to the treatment of acute disease. However, its stability might make it ideal as a prognostic tool and a more reliable marker of low‐grade, systemic, chronic inflammation. This claim is supported by the fact that suPAR is correlated with disease progression^19^, ^22^ and mortality^23^, ^38^, ^39^, ^40^ across diverse patient populations and diseases.

In our study cohort, the majority of deaths occurred within 100 days from the index admission (Figure 2). As stated above, suPAR has been shown to exhibit a delayed response to acute inflammation unlike traditional acute‐phase reactants such as CRP and IL‐6.^16^ Consequently, the suPAR levels, which were measured within 2 hours after admission, are likely to reflect the chronic inflammatory status of the patients in the study cohort rather than their acute inflammatory response. This finding suggests that individuals with a high chronic inflammatory burden (suPAR ≥6 ng/mL) may have a diminished capacity to tolerate and recover from acute illnesses, including hospitalization for AF. If this is the case, suPAR may indeed serve as a particularly valuable biomarker for predicting early mortality in acutely admitted AF patients, while its utility in forecasting long‐term survival for this population may be more limited. However, as the follow‐up period in our study was restricted to a maximum of 1 year, it remains uncertain whether the divergence in survival curves would widen over time.

The clinical consequences of elevated suPAR levels in patients hospitalized acutely with AF remain unknown—aside perhaps from being a predictor of worsened prognosis. To date, no drug has been used specifically to target inflammatory pathways in patients with AF. Future studies are warranted to study whether anti‐inflammatory treatment could reduce suPAR and ultimately improve clinical outcomes in AF.

Several limitations of this study must be acknowledged. The observational nature of our study does not allow us to imply a causal association between suPAR and mortality in AF patients. Although we adjusted for several potential confounding factors, there is a risk of type I and II errors. We cannot rule out the possibility that the association between suPAR levels and mortality in patients with AF is confounded by unaccounted comorbidities. Moreover, the cause of death was not available; hence, we could not report on cardiovascular mortality. Since admissions with AF in the cohort were extracted from the electronic patient record system using ICD‐10 codes, potential misclassification can occur of the AF diagnosis. However, our cohort's prevalence of AF aligns with other studies.^41^ Another limitation is the lack of data regarding changes in suPAR levels over time, which could provide insights to the mechanisms leading to mortality.

## 5 CONCLUSION

In patients acutely admitted to an ED with AF, the inflammatory biomarker suPAR, measured upon admission, was significantly associated with mortality. After adjustments for potential confounders, an increase in suPAR of 1 ng/mL was independently associated with a 12% increase in the risk of 1‐year all‐cause mortality.

## CONFLICT OF INTEREST STATEMENT

The authors declare no conflicts of interest.

## ETHICS STATEMENT

This study was carried out in accordance with the Code of Ethics of the World Medical Association (Declaration of Helsinki). The database and collection of clinical data were approved by the Danish Data Protection Agency (record no. P‐2020‐513) and by the Danish Patient Safety Authority (record no. 31–1521‐319).

## INFORMED CONSENT/CONSENT TO PUBLISH

Written informed consent was obtained from all individuals included in the study. Patients provided written informed consent regarding publishing the data.

## PERMISSION TO REPRODUCE MATERIAL FROM OTHER SOURCES

We will grant approval to reproduce the material upon reasonable request.

## Supporting information


Data S1.

## Data Availability

The data that support the findings of this study are available from the Department of Clinical Research, Copenhagen University Hospital, Amager and Hvidovre, Hvidovre, Denmark. Restrictions apply to the availability of these data, which were used under license for this study, and so are not publicly available. Research data will be shared upon reasonable request.
