# Supplementary material for: Soluble urokinase plasminogen activator receptor (suPAR) as a prognostic biomarker in acutely admitted patients with atrial fibrillation
Source: J Arrhythm. 2025 Apr 23;41(2):e70077. doi: 10.1002/joa3.70077 (PMC12017082; doi:10.1002/joa3.70077)
Supplement: Supplementary file 1 — Data S1. [file JOA3-41-e70077-s001.docx]

**Supplementary**

Supplementary, Figure 1. Histogram showing the frequency of suPAR levels in the cohort of patients admitted with AF

*
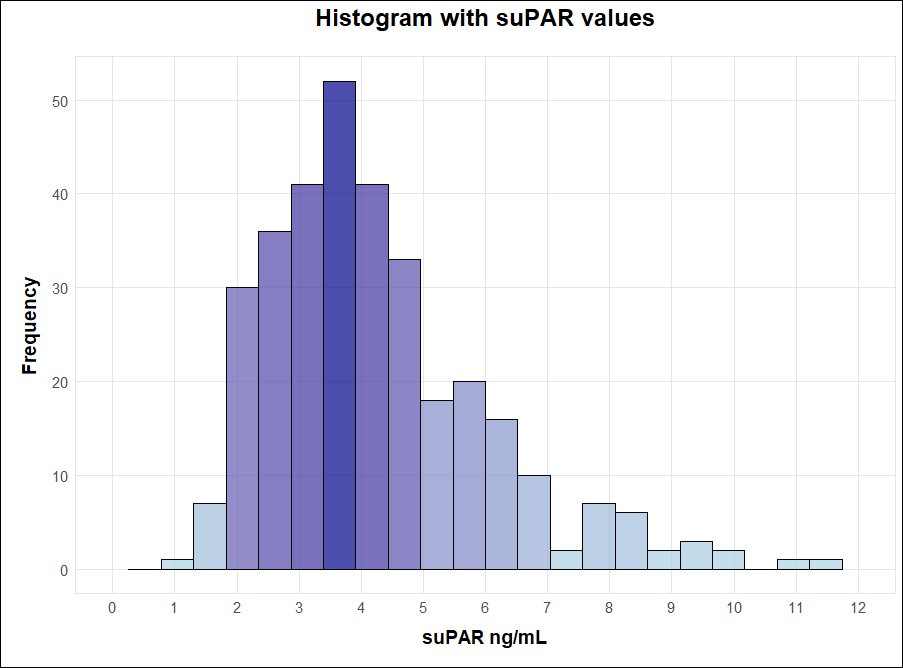
*

*suPAR: soluble urokinase plasminogen activator receptor*

Supplementary, Figure 2. Jitter plot showing the distribution of suPAR levels stratified according to the defined intervals


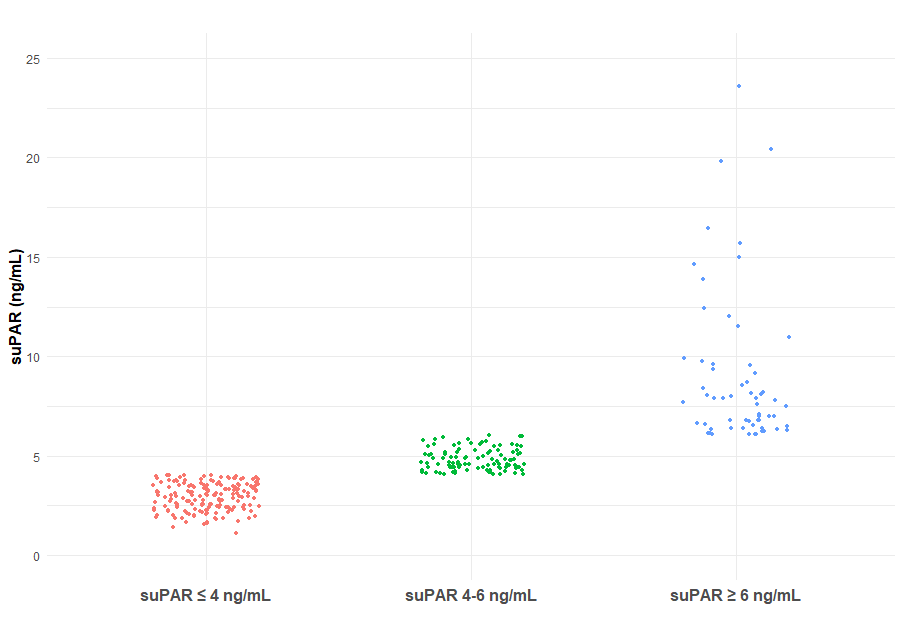


suPAR: soluble urokinase plasminogen activator receptor

Supplementary, Figure 3. Forrest plot showing the association between unadjusted 1-year mortality and suPAR stratifed into defined groups
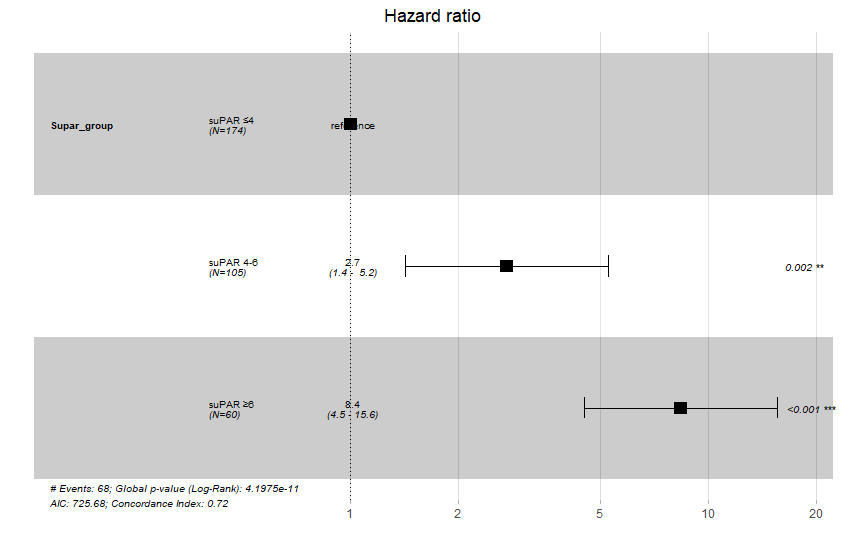


*suPAR: soluble urokinase plasminogen activator receptor*

Supplementary, Figure 4. Forrest plot showing the association between 1-year mortality and various variables (model 1).

*
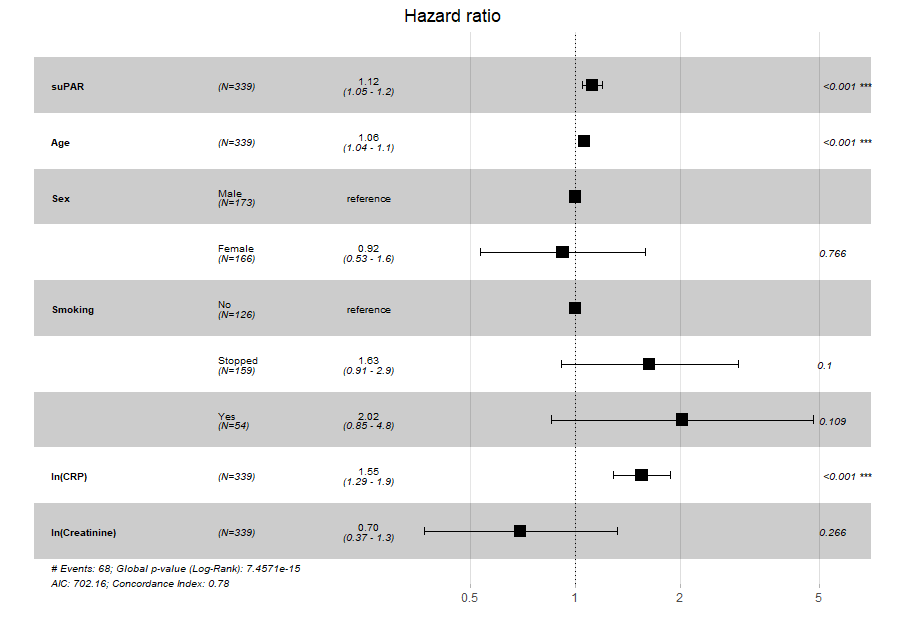
suPAR: soluble urokinase plasminogen activator receptor, ln(CRP): ln(C-reactive protein)*

*Supplementary, Figure 5. Forrest plot showing the association between 1-year mortality and various variables (model 2).*

*
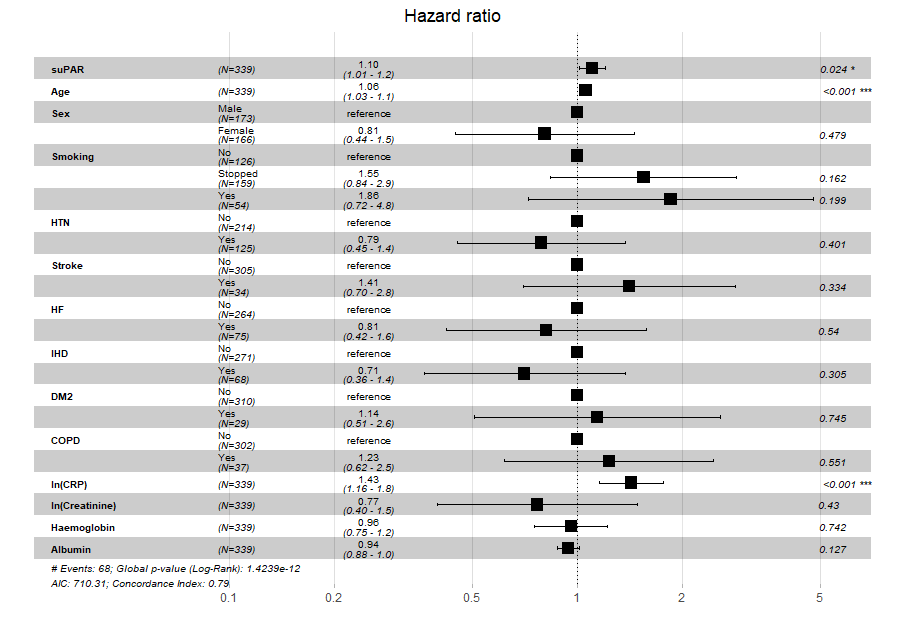
 suPAR: soluble urokinase plasminogen activator receptor, HTN: hypertension, HF: heart failure, IHD: ischemic heart disease; DM2: Type 2 Diabetes Mellitus, COPD: chronic obstructive lung disease, ln(CRP): ln(C-reactive protein)*

*Supplementary, Figure 6. Forest plot showing the association between 1-year mortality and various variables. Supar is stratified accoring to the defined intervals (model 3).*

*
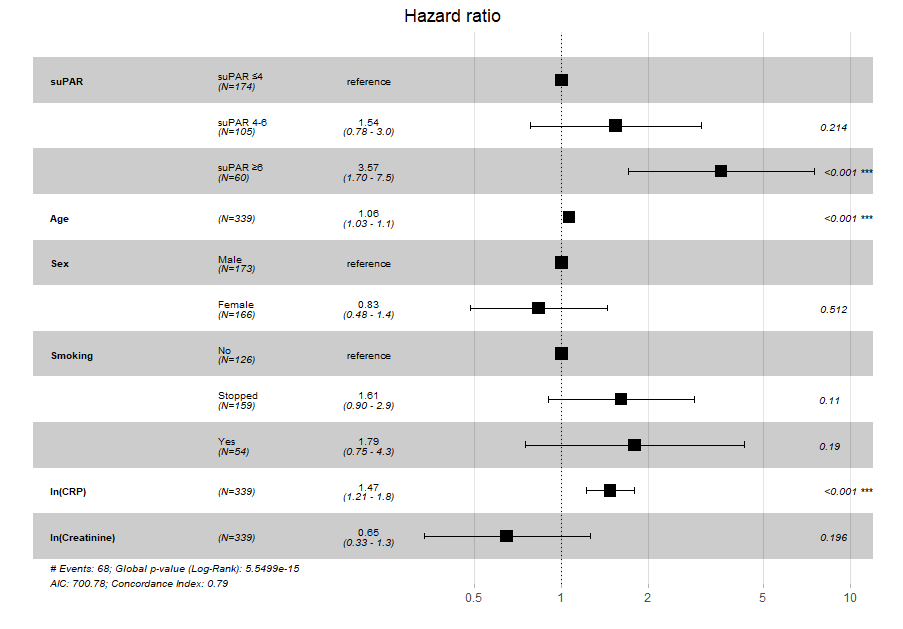
*

*suPAR: soluble urokinase plasminogen activator receptor ln(CRP): ln(C-reactive protein)*

Supplementary, Figure 7. Receiver operating characteristics (ROC) curves showing the performance of CRP and suPAR on the predicting of 1-year mortality.


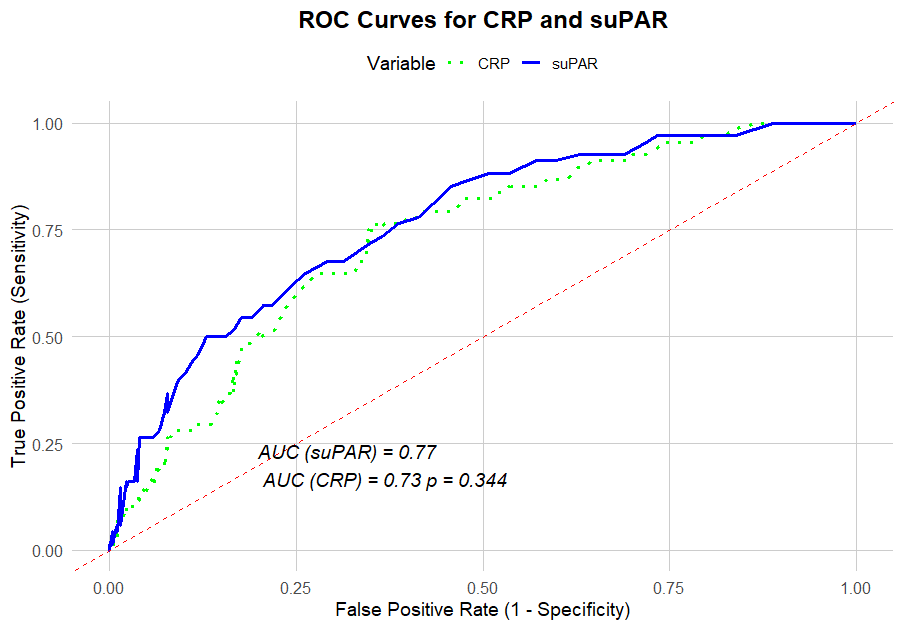


suPAR: soluble urokinase plasminogen activator receptor, CRP: C-reactive protein

Supplementary, Figure 8. Poisson regression showing the association between length of in- hospital stay (index admission) and various variables.

| Coefficients: |  |  |  |  |
| --- | --- | --- | --- | --- |
|  | Estimate | Std. Error | z value | p value |
| (Intercept) | 0.67 | 0.14 | 4.7 | <0.001 |
| SuPAR | 0.076 | 0.0063 | 11.9 | <0.001 |
| Age | 0.013 | 0.0019 | 6.8 | <0.001 |
| Sex (Female) | 0.096 | 0.48 | 2.00 | 0.045 |
| Smoking (quit) | -0.018 | 0.051 | -0.35 | 0.73 |
| Smoking (previous) | 0.66 | 0.064 | 10.4 | <0.001 |
| CRP | 0.0028 | <0.001 | 8.9 | <0.001 |
| Creatinine | 0.0012 | <0.001 | 5.7 | <0.001 |

*suPAR: soluble urokinase plasminogen activator receptor, CRP: C-reactive protein*

Supplementary, Figure 9. Poisson regression showing the association between readmission (within one year from index admission) and various variables.

| Coefficients: |  |  |  |  |
| --- | --- | --- | --- | --- |
|  | Estimate | Std. Error | z value | p value |
| (Intercept) | 0.81 | 0.24 | 3.4 | <0.001 |
| SuPAR | -0.0082 | 0.019 | -0.44 | 0.66 |
| Age | -0.0019 | 0.0033 | -0.55 | 0.58 |
| Sex (female) | 0.017 | 0.088 | 0.20 | 0.85 |
| Smoking(previous) | -0.14 | 0.088 | -1.6 | 0.12 |
| Smoking (current) | -2.6 | 0.13 | -2.03 | 0.042 |
| CRP | 0.0012 | <0.001 | 1.6 | 0.10 |
| Creatinine | <0.001 | <0.001 | 0.58 | 0.56 |

*suPAR: soluble urokinase plasminogen activator receptor, CRP: C-reactive protein*

Supplementary, Figure 10. Binary logistic regression showing the association between admission to the ICU (within one year from index admission) and various variables.

| Coefficients: |  |  |  |  |  |
| --- | --- | --- | --- | --- | --- |
|  | Estimate | Std. Error | z value | p value |  |
| (Intercept) | 3.1 | 1.6 | 1.9 | 0.056 |  |
| SuPAR | -0.067 | 0.81 | -0.81 | 0.42 |  |
| Age | 0.012 | 0.021 | 0.83 | 0.41 |  |
| Sex (female) | 0.29 | 0.56 | 0.52 | 0.60 |  |
| Smoking (quit) | -1.5 | 0.79 | -1.9 | 0.061 |  |
| Smoking (previous) | -1.1 | 0.95 | -1.2 | 0.25 |  |
| CRP | -0.0050 | 0.0034 | -1.5 | 0.14 |  |
| Creatinine | <0.001 | 0.0029 | 0.099 | 0.92 |  |

*suPAR: soluble urokinase plasminogen activator receptor, CRP: C-reactive protein*
